# Assessing the Relationship Between Vitiligo and Major Depressive Disorder Severity: Cross-Sectional Study

**DOI:** 10.2196/60686

**Published:** 2024-07-12

**Authors:** Amr Molla, Raed Jannadi, Hamza Alayoubi, Haya Altouri, Maryam Balkhair, Dareen Hafez

**Affiliations:** 1 Department of Medicine College of Medicine Taibah University Madinah Saudi Arabia; 2 Department of Family, Community Medicine, & Medical Education College of Medicine Taibah University Madinah Saudi Arabia; 3 Internship Unit College of Medicine Taibah University Madinah Saudi Arabia; 4 Department of Psychiatry Al-Amal Complex for Mental Health Madinah Saudi Arabia

**Keywords:** vitiligo, major depressive disorder (MDD), PHQ-9, Patient Health Questionnaire-9, depression severity, Saudi Arabia, cross-sectional study

## Abstract

**Background:**

Vitiligo, a common dermatological disorder in Saudi Arabia, is associated with significant psychological impacts. This study explores the relationship between vitiligo and the severity of major depressive disorder (MDD), highlighting the broader implications on mental health among affected individuals.

**Objective:**

We aim to assess the prevalence and predictors of depression among adult patients with vitiligo, and to examine the relationship between MDD severity and vitiligo.

**Methods:**

Using a cross-sectional design, the research used the vitiligo area severity index and the Patient Health Questionnaire-9 to measure the extent of vitiligo and depression severity, respectively. This study involved 340 diagnosed patients with vitiligo from various health care settings. Logistic and ordinal regression analysis were applied to evaluate the impact of sociodemographic variables and vitiligo types on MDD severity.

**Results:**

The prevalence of MDD was 58.8% (200/340) of participants. Depression severity varied notably: 18.2% (62/340) of patients experienced mild depression, 17.9% (61/340) moderate, 11.8% (40/340) moderately severe, and 10.9% (37/340) severe depression. Female patients had higher odds of severe depression than male patients (adjusted odds ratio [aOR] 3.14, 95% CI 1.93-5.1; *P*<.001). Age was inversely related to depression severity, with patients aged older than 60 years showing significantly lower odds (aOR 0.1, 95% CI 0.03-0.39; *P*<.001). Lower income was associated with higher depression severity (aOR 10.2, 95% CI 3.25-31.8; *P*<.001). Vitiligo types also influenced depression severity; vulgaris (aOR 5.3, 95% CI 2.6-10.9; *P*<.001) and acrofacial vitiligo (aOR 2.8, 95% CI 1.5-5.1; *P*<.001) were significantly associated with higher depression levels compared to focal vitiligo.

**Conclusions:**

The findings suggest that vitiligo contributes to an increased risk of severe depression, highlighting the need for integrated dermatological and psychological treatment approaches to address both the physical and mental health aspects of the disease.

## Introduction

Skin, the largest organ of the human body, serves as the visible exterior that covers internal structures. Vitiligo is a chronic, relapsing skin disorder characterized by well-defined milky-white, depigmented macules and patches resulting from the destruction of melanocytes [1]. This condition is often associated with other autoimmune disorders, particularly thyroid autoimmune diseases. Beyond the physical manifestations, vitiligo imposes a significant psychological burden due to its impact on cosmetic appearance, leading to potential stigmatization and misconceptions within social interactions. Consequently, individuals with vitiligo are at an elevated risk of developing major depressive disorder (MDD) [2].

MDD is marked by persistent sadness, loneliness, and disinterest, typically triggered by fear, trauma, or other significant stressors. This condition not only affects mood and interest but also impairs cognitive functions, influencing emotions, sleep, and appetite. These changes can alter behavior, making individuals appear irritable or despondent, with severe cases potentially leading to suicidal ideation [3]. The prevalence of MDD for life is 16.2% [4]. According to a meta-analysis study, the prevalence of depression among patients with vitiligo is 25.3% [5].

Patients with vitiligo often exhibit a dysregulated immune system, which may be exacerbated by concomitant depression. Clinical and animal studies suggest that depression can aggravate vitiligo, as both conditions share similar leukocyte signatures and inflammatory genetic mechanisms associated with systemic autoimmune inflammation. This overlap suggests a shared pathophysiological pathway, potentially increasing the risk of an inflammatory brain-skin axis, offering new insights into their bidirectional relationship and their classification within a socially stress-stigmatized model [6].

Recent studies have highlighted specific gene expression profiles associated with vitiligo, revealing significant molecular mechanisms underlying the condition. Changes in the expression of interleukin (IL)-10 family cytokines (IL26, IL-28A, IL28B, and IL29) and their receptor subunits (IL20RB, IL22RA2, and IL28RA), along with other genes related to melanocyte function such as *MDM1*, *IFNA1*, *IFNB1*, *IFNG*, and *ICAM1*, have been observed in the skin and peripheral blood mononuclear cells of patients with vitiligo. These genes are implicated in pathways regulating melanocyte survival, apoptosis, development, migration, and melanogenesis, suggesting their role in vitiligo pathogenesis [7]. Additionally, increased dopamine levels and altered expression of enzymes in the dopamine pathway, including DOPA decarboxylase, monoamine oxidase A, and monoamine oxidase B, have been noted in vitiligo patients’ skin and blood. This suggests that the dopamine pathway may influence melanogenesis directly or through the melanocortin pathway [8]. Furthermore, another study supports the role of IL-10 family cytokines in vitiligo pathogenesis, particularly emphasizing the involvement of IL-22. Altered expression patterns of IL20RB, IL22RA2, IL-28A, IL28B, IL28RA, *MDM1*, *IFNA1*, *IFNB1*, *IFNG*, and *ICAM1* in vitiligo skin and peripheral blood mononuclear cells further underscore their significance in the disease [9].

Global studies indicate that vitiligo significantly affects mental health, often leading individuals to self-isolation and avoidance of social gatherings, thereby severely affecting quality of life. Acceptance and active coping can mitigate stress and anxiety; however, the appearance-related impacts of vitiligo and MDD can lead to social withdrawal, sensitivity to perceived societal judgments, and overall deterioration in personal and professional life, culminating in diminished self-esteem and confidence [10,11].

Inspired by the PASI (psoriasis area and severity index), the vitiligo area severity index (VASI) uses hand units to quantify affected skin areas, where 1 hand unit approximates 1% of total body skin. The VASI score is calculated by multiplying the area of vitiligo (in hand units) by the degree of depigmentation within each measured patch [12].

This study aims to assess the prevalence and predictors of depression among adult patients with vitiligo. Moreover, examining the relationship between MDD severity and vitiligo, using the VASI and Patient Health Questionnaire-9 (PHQ-9) scales to assess the extent and severity of both conditions.

## Methods

### Study Design and Sample

This is a cross-sectional study aimed to investigate the relationship between MDD and vitiligo in Saudi Arabia. This study targeted adult patients diagnosed with vitiligo based on VASI and Wood’s lamp examination, which revealed depigmented patches or macules that occur at typical vitiligo sites. A total of 340 adult patients with vitiligo were selected by simple random sampling method for this study, which was conducted from April 2023 to April 2024 at participating hospitals across Saudi Arabia.

The inclusion and exclusion criteria for participation were as follows:

Inclusion criteria: (1) patients diagnosed with vitiligo, as determined by the VASI, (2) ages ranging from 18 to 85 years, (3) resident in Saudi Arabia during this study’s period, and (4) able to provide informed consent and complete this study’s assessments.

VASI score measures the extent and severity of vitiligo by evaluating the body’s surface area affected by vitiligo and quantifies the degree of skin depigmentation. The index divides the body into segments, with each segment’s depigmentation severity scored on a scale from 0% (no depigmentation) to 100% (complete depigmentation). The score for each segment is calculated by multiplying the affected body surface area percentage by the depigmentation level, which provides a comprehensive measure of the disease’s severity [13].

Exclusion criteria: (1) known preexisting mental health disorders before the diagnosis of vitiligo (eg, diagnosed MDD, bipolar disorder, and schizophrenia); (2) other forms of skin depigmentation not classified as vitiligo, such as albinism or chemical leukoderma; (3) cognitive impairments or any conditions that might hinder comprehension of the questionnaire or informed consent process; and (4) pregnant or lactating women, due to potential hormonal effects on skin condition and mood.

### Data Collection

Data were collected by using a web-based questionnaire conducted through social media of the patient contact number or email, phone call, or interview clinic during the period of this study. The questionnaire was divided into 2 parts. The first part captured sociodemographic information such as sex, age, marital status, nationality, job, and monthly income, while the second part consisted of the PHQ-9 to assess symptoms of depression.

### Study Variables

#### Independent Variable: Vitiligo Type

In this study, the independent variable was the type of vitiligo, categorized according to the revised classification from the Vitiligo Global Issues Consensus Conference (2012) [14]. This system classifies vitiligo into 3 primary clinical forms. Nonsegmental vitiligo encompasses generalized vitiligo (formerly known as vulgaris), acrofacial vitiligo with its subtype referred to as “lip-tip” vitiligo, and vitiligo universalis. Segmental vitiligo is characterized by a unilateral, asymmetric distribution. Unclassified vitiligo includes cases that do not evolve into either segmental or nonsegmental forms within a long period, such as focal vitiligo and single mucosal vitiligo affecting either genital areas or the oral cavity.

#### Dependent Variable: MDD

PHQ-9 score was used to assess the severity of depressive symptoms. The PHQ-9 is a clinician-administered instrument that screens for depression and grades symptom severity based on the criteria from the *DSM-IV* (*Diagnostic and Statistical Manual of Mental Disorders* [Fourth Edition]) [15,16]. Respondents rate how often they have experienced each of the 9 *DSM-IV* criteria for depression over the past 2 weeks on a scale from 0 (“not at all”) to 3 (“nearly every day”). The total possible score ranges from 0 to 27, with higher scores indicating greater depression severity. The depression severity categories are none to minimal depression (0-4), mild depression (5-9), moderate depression (10-14), moderately severe depression (15-19), and severe depression (20-27).

Further, 2 variables were created based on the PHQ-9 score. First, the depression severity variable was coded as follows: 0=non, 1=mild, 2=moderate, 3=moderately severe, and 4=severe depression. The binary depression variable was categorized into 2 groups whether the patient has depression (mild to severe) or not (none or minimal).

### Covariates

In this study, the covariates include gender, age, nationality, marital status, job, and monthly income. Gender was categorized as male or female. Age was divided into 3 groups: 18-25 years, 26-60 years, and older than 60 years, with the 26-60 age group serving as the reference category in multivariate analysis. Nationality was classified as either Saudi or non-Saudi. Income levels were categorized into 4 groups, ranging from less than US $810 per month for the low-income group to over US $5265 per month for the high-income group, with the moderately high-income group (ranging from US $2430 to US $5265 per month) used as the reference in regression analysis. Marital status was classified as single, married, or divorced, with married participants considered the reference group. Job status was divided into 3 categories: employed, unemployed, or student.

### Data Management and Analysis

Data were collected electronically and analyzed using SPSS software (version 29; IBM Corp). For bivariate analysis, the chi-square test was used to investigate the association between categorical variables. The Monte Carlo simulation method was applied when indicated as an alternative to the standard chi-square test. Logistic regression analysis was conducted to explain the variability of depression occurrence by including sex, age, nationality, marital status, income, employment status, and vitiligo types in the final model. Similarly, the same model was applied in ordinal logistic regression analysis to predict depression severity. A *P* value less than .05 was considered statistically significant.

### Ethical Considerations

This paper is original, unpublished, and not under consideration elsewhere. All content, unless cited, is based on our unique research. We adhered to Saudi ethical standards, obtaining Taibah University institutional review board approval (TU-039-22) on June 5, 2023, and participant consent, respecting confidentiality and the Declaration of Helsinki.

## Results

### Sociodemographic Characteristics

Table 1 shows the sociodemographic characteristics of this study’s sample stratified by depression status. The prevalence of depression among patients with vitiligo was 58.8%, with 200 of 340 participants having depression. A significant gender difference in depression prevalence was observed. Women showed a higher prevalence, with 109 of 156 (69.9%) women experiencing depression compared to 91 of 184 (49.5%) men, and this difference was statistically significant (*P*<.001). Moreover, age played a significant role in depression among patients with vitiligo. The 18- to 25-year age group had the highest depression rate, with 51 of 69 (73.9%) participants experiencing depression, followed by the 26- to 60-year age group with 146 of 248 (58.9%) participants experiencing depression, while those older than 60 years had a much lower rate of 3 of 23 (13%) participants experiencing depression (*P*<.001). Marital status was significantly associated with depression development, where divorced and single individuals were more likely to be depressed, with 34 of 41 (82.9%) divorced participants and 115 of 153 (75.2%) single participants experiencing depression, compared to 51 of 146 (34.9%; *P*<.001) married patients. Depression development varied significantly across income levels. The lowest income group had a higher depression proportion, with 181 of 253 (71.5%) participants experiencing depression. In contrast, individuals in the high-moderate income category showed a much lower depression prevalence, with 6 of 43 (14%) participants experiencing depression (*P*<.001; Table 1).

**Table 1:** Demographic and socioeconomic influences on depression status among patients with vitiligo.

Variables			Depression		*P* value	
		Total (N=340), n (%)	Yes (n=200), n (%)	No (n=140), n (%)		
**Gender**						<.001
	Male	184 (54.1)	91 (49.5)	93 (50.5)		
	Female	156 (45.9)	109 (69.9)	47 (30.1)		
**Age groups (years)**						<.001
	18-25	69 (20.3)	51 (73.9)	18 (26.1)		
	26-60	248 (72.9)	146 (58.9)	102 (41.1)		
	>60	23 (6.8)	3 (13)	20 (87)		
**Nationality**						.17
	Saudi	290 (85.3)	175 (60.3)	115 (39.7)		
	Non-Saudi	50 (14.7)	25 (50)	25 (25)		
**Marital status**						<.001
	Single	153 (45)	115 (75.2)	38 (24.8)		
	Divorced	41 (12.1)	34 (82.9)	7 (17.1)		
	Married	146 (42.9)	51 (34.9)	95 (65.1)		
**Income**						<.001^a^
	Low	253 (74.4)	181 (71.5)	72 (28.5)		
	Low-moderate	32 (9.4)	10 (31.3)	22 (68.8)		
	High-moderate	43 (12.6)	6 (14)	37 (86)		
	High	12 (3.5)	3 (25)	9 (75)		
**Job**						<.001
	Yes	128 (37.6)	46 (35.9)	82 (64.1)		
	No	183 (53.8)	133 (72.7)	50 (27.3)		
	Student	29 (8.5)	21 (72.4)	8 (27.6)		

^a^*P* value calculated by using the Monte Carlo simulation.

### Bivariate Association of Depression Severity With Sociodemographic Characteristics

Table 2 illustrates the bivariate association of depression severity and various sociodemographic characteristics of this study’s sample. Men were less likely to experience severe depression than women, with 2 of 184 (1.1%) men experiencing severe depression versus 35 of 156 (22.4%; *P*<.001) women. Furthermore, age group analysis reveals that most (20/23, 87%) of the older patients with vitiligo (>60 years) did not have depression, while the youngest age group (18-25 years) had more representation in the moderate and moderately severe categories, with 18 of 69 (26.1%) experiencing moderate depression and 12 of 69 (17.4%) experiencing moderately severe depression (*P*<.001). Marital status was significantly associated with depression severity, where divorced individuals exhibited higher rates of severe depression, with 13 of 41 (31.7%) experiencing severe depression compared to 22 of 153 (14.4%) singles and 2 of 146 (1.4%; *P*<.001) married individuals. Moreover, the low-income group notably had higher rates of moderate to severe depression compared to higher-income groups. For instance, in the low-income group, 91 of 253 (36%) participants experienced moderate to severe depression, whereas in the high-moderate income group, only 2 of 43 (4.7%) participants experienced moderate to severe depression (*P*<.001; Table 2).

**Table 2:** Variation in depression severity across demographic and socioeconomic characteristics of patients with vitiligo.

Variables					Depression severity												
			Total(N=340), n (%)		None (n=140), n (%)		Mild (n=62), n (%)		Moderate (n=61), N (%)		Moderately severe (n=40), N (%)		Severe (n=37), N (%)		*P* value		
**Gender**																	<.001
	Male	184 (54.1)		93 (50.5)		44 (23.9)		32 (17.4)		13 (7.1)		2 (1.1)					
	Female	156 (45.9)		47 (30.1)		18 (11.5)		29 (18.6)		27 (17.3)		35 (22.4)					
**Age groups (years)**																	<.001^a^
	18-25	69 (20.3)		18 (26.1)		14 (20.3)		18 (26.1)		12 (17.4)		7 (10.1)					
	26-60	248 (72.9)		102 (41.1)		48 (19.4)		40 (16.1)		28 (11.3)		30 (12.1)					
	>60	23 (6.8)		20 (87)		0 (0)		3 (13)		0 (0)		0 (0)					
**Nationality**																	.11
	Saudi	290 (85.3)		115 (39.7)		49 (16.9)		55 (19)		36 (12.4)		35 (12.1)					
	Non-Saudi	50 (14.7)		25 (50)		13 (26)		6 (12)		4 (8)		2 (4)					
**Marital status**																	<.001^a^
	Single	153 (45)		38 (24.8)		36 (23.5)		36 (23.5)		21 (13.7)		22 (14.4)					
	Divorced	41 (12.1)		7 (17.1)		2 (4.9)		7 (17.1)		12 (29.3)		13 (31.7)					
	Married	146 (42.9)		95 (65.1)		24 (16.4)		18 (12.3)		7 (4.8)		2 (1.4)					
**Income**																	<.001^a^
	Low	253 (74.4)		72 (28.5)		54 (21.3)		52 (20.6)		39 (15.4)		36 (14.2)					
	Low-moderate	32 (9.4)		22 (68.8)		3 (9.4)		5 (15.6)		1 (3.1)		1 (3.1)					
	High-moderate	43 (12.6)		73 (86)		4 (9.3)		2 (4.7)		0 (0)		0 (0)					
	High	12 (3.5)		9 (75)		1 (8.3)		2 (16.7)		0 (0)		0 (0)					
**Job**																	<.001^a^
	Yes	128 (37.6)		82 (64.1)		20 (15.6)		15 (11.7)		6 (4.7)		5 (3.9)					
	No	183 (53.8)		50 (27.3)		34 (18.6)		39 (21.3)		29 (15.8)		31 (16.9)					
	Student	29 (8.5)		8 (27.6)		8 (27.6)		7 (24.1)		5 (17.2)		1 (3.4)					

^a^*P* value calculated by using the Monte Carlo simulation.

### Association of Vitiligo Types With Depression Severity

Vitiligo types varied significantly in their association with depression severity. Acrofacial vitiligo was the most common type, affecting 165 (48.5%) patients. This group showed a higher proportion of moderate to severe depression, with 73 of 165 (44.2%) patients experiencing moderate to severe depression compared to a more localized focal vitiligo, with 16 of 65 (24.7%) patients experiencing moderate to severe depression. Vulgaris vitiligo was observed in 73 (21.5%) patients and revealed the highest proportion of moderate to severe depression, with 42 of 73 (57.6%) patients experiencing moderate to severe depression. On the other hand, universalis vitiligo appeared in 11 (3.2%) patients, with a majority, 10 of 11 (90.9%) patients, having no depression, and the remaining proportion, 1 of 11 (9.1%) patients, experienced mild depression. Similarly, genital vitiligo affected 16 (4.7%) patients, mostly with no depression, with 10 of 16 (62.5%) patients having no depression, and lesser extents of mild depression, with 3 of 16 (18.8%) patients, moderate depression with 2 of 16 (12.5%) patients, and moderately severe depression, with 1 of 16 (6.3%) patients (Table 3).

**Table 3:** Correlation between vitiligo types and depression severity among patients.

		Depression severity						Total (N=340), n (%)		*P* value	
		None (n=140), n (%)	Mild (n=62), n (%)	Moderate (n=61), n (%)	Moderately severe (n=40), n (%)	Severe (n=37), n (%)					
**Vitiligo types**											.01^a^
	Acrofacial	61 (37)	31 (18.8)	34 (20.6)	20 (12.1)	19 (11.5)	165 (48.5)				
	Vulgaris	24 (32.9)	7 (9.6)	18 (24.7)	14 (19.2)	10 (13.7)	73 (21.5)				
	Focal	33 (50.8)	16 (24.6)	7 (10.8)	4 (6.2)	5 (7.7)	65 (19.1)				
	Universalis	10 (90.9)	1 (9.1)	0 (0)	0 (0)	0 (0)	11 (3.2)				
	Segmental	2 (20)	4 (40)	0 (0)	1 (10)	3 (30)	10 (2.9)				
	Genital	10 (62.5)	3 (18.8)	2 (12.5)	1 (6.3)	0 (0)	16 (4.7)				

^a^*P* value calculated by using the Monte Carlo simulation.

### Logistic Regression Analysis of Depression Risk Factors

Logistic regression analysis indicates that the age group over 60 years is significantly less likely to develop MDD compared to the reference group (aged 26-60 years), with an adjusted odds ratio (aOR) of 0.12 (95% CI 0.03-0.48; *P*=.002). Women did not have a statistically significant higher risk of developing depression than men, with an aOR of 1.29 (95% CI 0.7-2.39; *P*=.42). Individuals with a low income had a significantly higher risk of developing MDD, with an aOR of 9.5 (95% CI 2.9-30.9; *P*<.001) compared to the moderately high-income reference group. Additionally, single patients had an aOR of 2.78 (95% CI 1.29-5.98; *P*=.01), and divorced individuals had an aOR of 3.86 (95% CI 1.28-11.67; *P*=.02) of having depression compared to married patients. Compared to localized focal vitiligo, segmental vitiligo showed the highest risk of depression development, with an aOR of 6.37 (95% CI 1.04-38.8; *P*=.045), followed by vulgaris and acrofacial types that showed significantly increased risks, with aORs of 3.5 (95% CI 1.46-8.38; *P*=.005) and 2.45 (95% CI 1.2-4.98; *P*=.01), respectively. In contrast, universalis vitiligo was associated with a significantly lower depression risk, with an aOR of 0.07 (95% CI 0.01-0.59; *P*=.02; Table 4).

**Table 4:** Logistic regression analysis of study variables influencing major depressive disorder development among patients with vitiligo.

Variables		aOR^a^ (95% CI)	*P* value
**Age groups (years)**			
	26-60 (reference)	1	—^b^
	18-25	0.67 (0.27-1.65)	.38
	>60	0.12 (0.03-0.48)	.002
**Gender**			
	Male (reference)	1	—
	Female	1.29 (0.7-2.39)	.42
**Income**			
	Moderately high (reference)	1	—
	High	2.95 (0.51-17.1)	.23
	Moderate	2.61 (0.78-8.78)	.12
	Low	9.5 (2.9-30.9)	<.001
**Marital status**			
	Married (reference)	1	—
	Single	2.78 (1.29-5.98)	.01
	Divorced	3.86 (1.28-11.67)	.02
**Vitiligo types**			
	Focal (reference)	1	—
	Universalis	0.07 (0.01-0.59)	.02
	Genital	1.24 (0.29-5.27)	.77
	Acrofacial	2.45 (1.2-4.98)	.01
	Vulgaris	3.5 (1.46-8.38)	.005
	Segmental	6.37 (1.04-38.8)	.045

^a^aOR was calculated by including age, gender, nationality, marital status, income, job, and vitiligo types.

^b^Not applicable.

### Ordinal Logistic Regression Analysis of Depression Severity

Table 5 demonstrates the ordinal logistic regression of depression severity by modeling depression in an ascending severity direction ranging from minimal to severe depression. Gender significantly influences the severity of depression, with women having a higher risk (aOR 3.14, 95% CI 1.93-5.1; *P*<.001) than men. Moreover, the age group of >60 years has a significantly lower risk of developing severe depression than the reference group (aged 26-60 years; aOR 0.1, 95% CI 0.03-0.39; *P*<.001). Being divorced is associated with a higher risk of having severe depression (aOR 5.8, 95% CI 2.6-12.9; *P*<.001) than married patients. Low-income patients were linked to a higher severity of depression, showing an aOR of 10.2 (95% CI 3.25-31.8; *P*<.001), than the moderately high-income group. The type of vitiligo shows a significant role in depression severity, with vulgaris (aOR 5.3, 95% CI 2.6-10.9; *P*<.001) and acrofacial (aOR 2.8, 95% CI 1.5-5.1; *P*<.001) types associated with a higher depression severity than focal vitiligo. On the other hand, universalis vitiligo shows a significantly lower association with severe depression (aOR 0.05, 95% CI 0.01-0.5; *P*=.01; Table 5).

**Table 5:** Ordinal logistic regression of factors affecting depression severity in patients with vitiligo.

Variables		aOR^a^ (95% CI)	*P* value
**Age groups (years)**			
	26-60 (reference)	1	—^b^
	18-25	0.97 (0.51-1.86)	.93
	>60	0.1 (0.03-0.39)	<.001
**Gender**			
	Male (reference)	1	—
	Female	3.14 (1.93-5.1)	<.001
**Income**			
	Moderately high (reference)	1	—
	High	4.1 (0.71-23)	.12
	Moderate	3.1 (0.91-10.6)	.07
	Low	10.2 (3.25-31.8)	<.001
**Marital status**			
	Married (reference)	1	—
	Single	2.7 (1.5-5.1)	.001
	Divorced	5.8 (2.6-12.9)	<.001
**Vitiligo types**			
	Focal (reference)	1	—
	Universalis	0.05 (0.01-0.5)	.01
	Acrofacial	2.8 (1.5-5.1)	<.001
	Vulgaris	5.3 (2.6-10.9)	<.001
	Segmental	4.4 (1.2-15.9)	.02
	Genital	1.7 (0.5-6.2)	.43

^a^aOR was calculated by including age, gender, nationality, marital status, income, job, and vitiligo types.

^b^Not applicable.

## Discussion

### Principal Findings

This study quantitatively assessed the prevalence and predictors of depression among patients with vitiligo in Saudi Arabia, using the VASI and PHQ-9. Our findings revealed a significant association between vitiligo and increased severity of MDD, with a prevalence of 58.8% (200/340) participants among the vitiligo population, substantially higher than the 16.2% lifetime prevalence reported for the general population [3,4]. Notably, MDD exhibits a higher severity among certain subgroups within the patients with vitiligo population. Specifically, women, divorced individuals, and those with lower income levels tend to experience more severe forms of depression. Additionally, the type of vitiligo also influences the severity of MDD, with acrofacial and vulgaris vitiligo associated with higher depression levels. These insights are crucial for tailoring more effective, targeted interventions for these vulnerable subgroups.

This study’s strengths include the use of validated tools such as the VASI and the PHQ-9, which enhance the reliability of our depression severity assessments. Additionally, our significant sample size and random sampling methodology provide a robust statistical basis for generalization within the target population. In addition, the PHQ-9 was administered by professional psychiatrists to assess MDD severity. However, this study also faces limitations. Being a cross-sectional study, it does not support causal inferences between vitiligo and the onset of MDD. Our findings might also not apply to other regions or ethnic groups since this study was geographically confined to Saudi Arabia. Moreover, excluding individuals with preexisting mental health conditions might lead to an underestimation of the actual psychological impact of vitiligo, as it does not consider those who might have developed MDD before the onset of vitiligo.

The observed high prevalence of MDD among patients with vitiligo supports the hypothesis that chronic skin diseases are significantly associated with psychiatric morbidities. This correlation is likely due to the visible and stigmatizing nature of vitiligo, which can lead to social withdrawal and significant psychological stress, thereby increasing the risk of depression. The results are consistent with findings from similar studies, which reported depression prevalence rates of 80% and 51.5% respectively [17,18], emphasizing the need for a multidisciplinary approach to managing patients with vitiligo, considering both dermatological and psychological aspects.

The findings from this study are primarily applicable to the adult population with vitiligo in Saudi Arabia. While these results provide valuable insights into the psychological impact of vitiligo, caution should be used when generalizing to populations in different settings or with different cultural backgrounds. Further research in varied demographic and ethnic groups is necessary to understand fully the global implications of these findings.

Recent studies have identified genetic associations related to immune-regulating genes in MDD. A case-control genetic association study involving the *IKBKE* gene, which encodes the IKKε protein involved in innate immunity and proinflammatory responses, revealed significant associations between *IKBKE* single-nucleotide polymorphism and MDD, as well as suggestive associations with panic disorder [19]. Additionally, polymorphisms in the limbic system-associated membrane protein (*LSAMP*) gene have shown strong associations with MDD and suggestive associations with panic disorder, suggesting a potential role for *LSAMP* in mood and anxiety disorders [20]. These genetic findings are particularly relevant to the immune dysregulation observed in patients with vitiligo, where immune-related genes play a crucial role in the pathogenesis of the disease. The proinflammatory properties of the *IKBKE* gene and its involvement in innate immunity could provide a mechanistic link between the immune responses in vitiligo and the increased susceptibility to mood disorders such as MDD. Similarly, the *LSAMP* gene’s association with mood disorders highlights the potential overlap in genetic pathways that may contribute to both vitiligo and MDD. The shared genetic markers and pathways between vitiligo and MDD suggest that immune dysregulation may be a common underlying factor. For instance, the involvement of proinflammatory cytokines and immune-modulating genes in both conditions underscores the importance of understanding how immune system alterations can influence both skin pathology and psychiatric outcomes. Further research into these shared genetic and molecular pathways could provide deeper insights into the comorbidity of vitiligo and mood disorders, potentially leading to more integrated therapeutic approaches targeting both the immune system and mental health.

Future research should use longitudinal designs to explore the causal relationships between vitiligo and depression. Studies testing the effectiveness of integrated treatment approaches for the physical and psychological aspects of vitiligo would also be beneficial. Expanding research to include diverse populations can help determine the broader applicability of these findings and explore cultural influences on the psychological impacts of vitiligo.

### Conclusion

In summary, this cross-sectional study has highlighted a significant association between vitiligo and the severity of MDD among patients, with a notably high prevalence of depression observed. The findings underscore the profound psychological impact of vitiligo, reinforcing the need for comprehensive treatment approaches that address both the dermatological and psychological aspects of the disorder. Future research should focus on longitudinal studies to explore the causative mechanisms between vitiligo and depression and evaluate the effectiveness of integrated treatment strategies.

## Abbreviations

aOR: adjusted odds ratio
DSM-IV: Diagnostic and Statistical Manual of Mental Disorders (Fourth Edition)
IL: interleukin
LSAMP: limbic system-associated membrane protein
MDD: major depressive disorder
PASI: psoriasis area and severity index
PHQ-9: Patient Health Questionnaire-9
VASI: vitiligo area severity index

## Data Availability

The data that support the findings of this research are available from the corresponding author upon reasonable request. Due to legal and ethical considerations, the data cannot be made publicly available. Requests for data access should be directed to AM.

## References

[1] Wolff K, Goldsmith LA, Katz S, Gilchrest BA, Paller AS, Leffell DJ (2008). Fitzpatrick's dermatology in general medicine: mcgraw-hill New York; 2008.

[2] Simons RE, Zevy DL, Jafferany M (2020). Psychodermatology of vitiligo: psychological impact and consequences. Dermatol Ther.

[3] Kessler RC, Berglund P, Demler O, Jin R, Koretz D, Merikangas KR, Rush AJ, Walters EE, Wang PS, National Comorbidity Survey Replication (2003). The epidemiology of major depressive disorder: results from the National Comorbidity Survey Replication (NCS-R). JAMA.

[4] Vallerand IA, Lewinson RT, Parsons LM, Hardin J, Haber RM, Lowerison MW, Barnabe C, Patten SB (2019). Vitiligo and major depressive disorder: a bidirectional population-based cohort study. J Am Acad Dermatol.

[5] Lai YC, Yew YW, Kennedy C, Schwartz RA (2017). Vitiligo and depression: a systematic review and meta-analysis of observational studies. Br J Dermatol.

[6] Otte C, Gold SM, Penninx BW, Pariante CM, Etkin A, Fava M, Mohr DC, Schatzberg AF (2016). Major depressive disorder. Nat Rev Dis Primers.

[7] Reimann E, Kingo K, Karelson M, Reemann P, Loite U, Sulakatko H, Keermann M, Raud K, Abram K, Vasar E, Silm H, Kõks S (2012). The mRNA expression profile of cytokines connected to the regulation of melanocyte functioning in vitiligo skin biopsy samples and peripheral blood mononuclear cells. Hum Immunol.

[8] Reimann E, Kingo K, Karelson M, Reemann P, Loite U, Keermann M, Abram K, Vasar E, Silm H, Kõks S (2012). Expression profile of genes associated with the dopamine pathway in vitiligo skin biopsies and blood sera. Dermatology.

[9] Reimann E, Kingo K, Karelson M, Reemann P, Vasar E, Silm H, Kõks S (2014). Whole transcriptome analysis (RNA Sequencing) of peripheral blood mononuclear cells of vitiligo patients. Dermatopathology (Basel).

[10] Nguyen CM, Beroukhim K, Danesh MJ, Babikian A, Koo J, Leon A (2016). The psychosocial impact of acne, vitiligo, and psoriasis: a review. Clin Cosmet Investig Dermatol.

[11] Salman A, Kurt E, Topcuoglu V, Demircay Z (2016). Social anxiety and quality of life in vitiligo and acne patients with facial involvement: a cross-sectional controlled Study. Am J Clin Dermatol.

[12] Dai YX, Tai YH, Chang YT, Chen TJ, Chen MH (2020). Association between major depressive disorder and subsequent autoimmune skin diseases: a nationwide population-based cohort study. J Affect Disord.

[13] Komen L, da Graça V, Wolkerstorfer A, de Rie MA, Terwee CB, van der Veen JPW (2015). Vitiligo area scoring index and vitiligo European task force assessment: reliable and responsive instruments to measure the degree of depigmentation in vitiligo. Br J Dermatol.

[14] Ezzedine K, Lim HW, Suzuki T, Katayama I, Hamzavi I, Lan CCE, Goh BK, Anbar T, Silva de Castro C, Lee AY, Parsad D, van Geel N, Le Poole IC, Oiso N, Benzekri L, Spritz R, Gauthier Y, Hann SK, Picardo M, Taieb A, Vitiligo Global Issue Consensus Conference Panelists (2012). Revised classification/nomenclature of vitiligo and related issues: the vitiligo global issues consensus conference. Pigment Cell Melanoma Res.

[15] Kroenke K, Spitzer RL, Williams JB (2001). The PHQ-9: validity of a brief depression severity measure. J Gen Intern Med.

[16] AlHadi AN, AlAteeq DA, Al-Sharif E, Bawazeer HM, Alanazi H, AlShomrani AT, Shuqdar RM, AlOwaybil R (2017). An arabic translation, reliability, and validation of patient health questionnaire in a Saudi sample. Ann Gen Psychiatry.

[17] Nasser MAEM, El Tahlawi SMR, Abdelfatah ZA, Soltan MR (2021). Stress, anxiety, and depression in patients with vitiligo. Middle East Curr Psychiatry.

[18] Alkhowailed M, Alotaibi HM, Alshwieer MA, Alazmi AK, Alotaibi NM, Alotaibi AF (2023). The psychological impact of vitiligo in Saudi Arabia. Cureus.

[19] Traks T, Koido K, Balõtšev R, Eller T, Kõks S, Maron E, Tõru I, Shlik J, Vasar E, Vasar V (2015). Polymorphisms of IKBKE gene are associated with major depressive disorder and panic disorder. Brain Behav.

[20] Koido K, Traks T, Balõtšev R, Eller T, Must A, Koks S, Maron E, Tõru I, Shlik J, Vasar V, Vasar E (2012). Associations between LSAMP gene polymorphisms and major depressive disorder and panic disorder. Transl Psychiatry.

